# What Is Known about Community Pharmacy-Based Take-Home Naloxone Programs and Program Interventions? A Scoping Review

**DOI:** 10.3390/pharmacy9010030

**Published:** 2021-02-02

**Authors:** Ashley Cid, George Daskalakis, Kelly Grindrod, Michael A. Beazely

**Affiliations:** School of Pharmacy, University of Waterloo, 10 Victoria St S A, Kitchener, ON N2G 1C5, Canada; Ashley.cid@uwaterloo.ca (A.C.); george.daskalakis@uwaterloo.ca (G.D.); mbeazely@uwaterloo.ca (M.A.B.)

**Keywords:** naloxone, harm reduction, community pharmacy

## Abstract

A variety of new sources describing community pharmacy-based take-home naloxone (THN) programs have emerged recently in the literature. There is a need to define the types of take-home naloxone programs being offered to support future research designs in implementing and evaluating standardized programs that fill pharmacist and patient knowledge gaps and lift current barriers for optimal community pharmacy naloxone provision. The objective of this paper is to summarize the literature on community pharmacy-based THN programs, including specific program interventions used to increase naloxone dispensing, naloxone availability and dispensing patterns, facilitators and barriers for the THN programs, and knowledge gaps. Online databases such as PubMed, EMBASE, Scopus, and International Pharmaceutical Abstracts (IPA) and a search of the grey literature were used to identify eligible sources. Sources were screened by two reviewers for eligibility in COVIDENCE software. Both reviewers compared screening results and resolved conflicts through discussion. A data extraction form for all identified full texts was completed by both reviewers and results were compiled through reviewer discussion. Fifty-two sources met the eligibility criteria. The top three barriers identified were: cost/coverage of naloxone, stigma, and education/training for pharmacists. THN program interventions included screening tools, checklists, pocket cards, patient brochures, and utilizing the pharmacy management system to flag eligible patients. Patient knowledge gaps included naloxone misinformation and lack of awareness, while pharmacists demonstrated administrative, clinical, and counselling knowledge gaps. Naloxone availability was found to be highly variable, where independent and rural pharmacies were less likely to stock or dispense naloxone. Further, pharmacies located in districts with higher rates of opioid overdose deaths and lower household income were also less likely to have naloxone available. This review identified multiple new programs, showcasing that the implementation and evaluation of THN programs are an expanding area of research. Future research should focus on implementing and evaluating a THN program through a randomized controlled trial design that incorporates solutions for the barriers and knowledge gaps identified in this study.

## 1. Introduction

The opioid crisis is growing every year. In Canada, more than 16,000 opioid-related deaths have occurred since January 2016 [1], and over 60,000 drug overdose deaths occurred in 2018 in the United States (US) [2]. These deaths are impacting national life expectancy rates [3], and opioids have now surpassed motor vehicle accidents as the leading cause of accidental death [4]. Naloxone, a temporary antidote for an opioid overdose, can be administered to those experiencing opioid overdose-related symptoms such as respiratory depression [3]. Naloxone was initially used only in hospital settings to reverse opioid toxicity but has recently been offered in community pharmacies. For example, in 2016, Health Canada removed naloxone from its prescription drug list, allowing naloxone to be dispensed by a community pharmacist without a prescription [5]. As of 2019, naloxone is also available in all community pharmacies in the US, but each state has different laws for naloxone access, such as standing orders, dispensing without a prescription, or pharmacist prescribing [6].

As one of the most accessible healthcare providers, pharmacists are ideally positioned to play a role in the opioid crisis by providing naloxone to patients who may be at risk of overdose or who may be in a position to help if they come into contact with someone who is experiencing an overdose [7]. Take-home naloxone (THN) programs are community pharmacy programs that allow for the provision of naloxone to eligible patients by registered pharmacists. The first THN programs emerged in the late 1990s and, since then, there have been a vast array of implementation and evaluation efforts to measure the success of these programs [8,9,10]. Different countries, regions, states, or healthcare areas which may be governed by different legislation may result in differences in supply. For example, naloxone kits are theoretically available in all community pharmacies in Canada based on its removal from the prescription drug list, while in some states in the US, pharmacists can independently prescribe and dispense naloxone through standing orders, where, without a standing order, pharmacists alone cannot initiate a prescription for naloxone without it being written by a physician [5,6]. Many variations exist within community pharmacies with respect to THN access, knowledge, and willingness to obtain or train on how to use naloxone [11]. Most pharmacy-based THN programs in Canada or the US are not mandatory and there is a considerable variability in the number of pharmacies who stock and/or dispense naloxone [11]. For example, in 2018, just over half of all pharmacies in Ontario dispensed naloxone, and a third of the kits dispensed were from only 1% of all pharmacies in large urban centers [12]. This demonstrates that there is a need for increased and equitable naloxone distribution through community pharmacy THN programs.

Two scoping reviews and one systematic review have been identified for studying pharmacy naloxone provision. In 2016, Nielsen and Van Hout analyzed the supply of naloxone in community pharmacies [13]. They found that the key barriers for pharmacy naloxone provision were identifying patient populations, supply systems, legal issues, and the training of pharmacists and patients [13]. In 2019, Muzyk et al. published a scoping review about pharmacists’ attitudes toward dispensing naloxone and other opioid use disorders [14]. They found that pharmacists generally have positive attitudes for patients at risk for opioid overdose or who have an opioid use disorder [14]. The main barriers mentioned for the implementation of dispensing naloxone include education and training, workflow, and management support [14]. Additionally, Thakur et al. published a systematic review on pharmacist roles, training, and perceived barriers in naloxone dispensing in 2020; however, the literature search was conducted between 2013 and 2018 [15]. Thakur et al. described that a key barrier to pharmacist naloxone dispensing was pharmacist training on identifying and educating patients at risk of an overdose [15]. They indicated that there was a need for an in-depth understanding of pharmacist perspectives on barriers to coordinate tailored approaches for increasing pharmacists’ confidence in naloxone dispensing [15]. A systematic review from 2014 discussing the effectiveness of community THN programs was also identified [10]. Clark et al. indicated that while it is hard to measure the efficacy of THN programs as no randomized controlled studies have been conducted, THN programs are continuing to increase in response to the rapidly increasing opioid overdose rates [10]. Therefore, only Clark et al. and Nielson and Van Hout specifically discussed what is known about THN programs, including their interventions, while Muzyk et al. focused on pharmacist attitudes and Thakur et al. focused on pharmacist roles and perceived barriers.

It is unclear what is currently known about community pharmacy-based THN programs and their program interventions, as no recent literature reviews specifically discussing the implementation of THN programs and their program interventions were identified since Clark et al.’s systematic review in 2014 and Nielson and Van Hout’s review in 2016 [10,13]. There are a multitude of studies reporting THN programs and their program interventions that have been recently published in 2019 and 2020, and a scoping review is needed to update the types of THN programs and their interventions because it allows for the potential for program expansion or development and program evaluation. Understanding current barriers and facilitators for existing THN programs and the knowledge gaps of pharmacists and patients is critical in ensuring the success of future community pharmacy take-home naloxone programs and the expansion of naloxone provision. With that in mind, the following research question was formulated to conduct a scoping review of the existing literature: what is known about pharmacy-based take-home naloxone programs? The scoping review was conducted with the objective of summarizing the literature on current community pharmacy-based take-home naloxone (THN) programs, including program interventions, naloxone availability, and dispensing patterns. This review also identifies any facilitators, barriers, and knowledge gaps regarding pharmacy-based THN programs from the patient and pharmacy team’s perspective.

## 2. Materials and Methods

The Arksey and O’Malley framework and PRISMA guidelines were used to guide the methodology of this scoping review [16,17]. Studies were included if they described a naloxone program based in a community pharmacy and were written in English or French. All peer-reviewed sources, such as quantitative or qualitative journal articles, conference proceedings, editorials, textbook chapters, and grey literature sources such as websites, were eligible to be included in the scoping review. There were no limits with respect to time frame in the search strategy as the authors wanted to capture all community pharmacy-based THN programs offered. Sources were excluded if they did not meet the inclusion criteria mentioned above, or the research was still in progress. Further reasons for exclusion were that the study was not talking about an existing THN program but rather a potential program, the THN program was not based in a community pharmacy, the focus of the paper was not about naloxone, or the paper only focused on talking about the role of the pharmacist in the opioid crisis.

To identify potentially relevant documents, the following databases were searched: PubMed, EMBASE, Scopus, and International Pharmaceutical Abstracts (IPA). The grey literature search included search engines such as Google Scholar and Google, as well as government and provincial or state pharmacy organization websites. The search strategies were created by AC and GD and were reviewed by an experienced librarian (see Appendix A for full search strategy example). The search strategies were further refined through team discussion as per the librarian’s suggestions. All search strategies except for grey literature were conducted by GD on 1 June 2020; the grey literature search was conducted by GD between 6 and 8 July 2020.

All sources including grey literature were exported into COVIDENCE (www.covidence.org, Melbourne AU) for review. Duplicates were automatically removed by the COVIDENCE software. AC and GD reviewed the sources individually, and manually removed any duplicates not automatically removed by COVIDENCE. AC and GD independently reviewed the titles, abstracts, and full-text publications, sequentially. AC reviewed the reference lists of all included sources after the full-text review was completed, to determine if any additional sources could be included. A pilot test of inclusion during the title and abstract reviewing stage occurred with the first 50 studies to test the inclusion criteria. The reviewers discussed any discrepancies identified by COVIDENCE at the title and abstract reviewing stage and at the full-text publication reviewing stage. If a discrepancy could not be resolved, a third reviewer, KG, reviewed the discrepancy and made a decision. No data were required to be obtained from any of the study investigators.

A data charting form was developed by AC and GD using the Arksey and O’Malley framework to determine which variables to extract; key finding fields were added to the form based on the study objectives [16]. The two reviewers independently charted the data using an online Google Form (see Appendix B) that then uploaded both reviewers’ responses to a Microsoft Excel sheet for compilation. Two sequential pilot tests of 10 articles using the data extraction form occurred. For each iteration, AC and GD discussed the results and clarified any discrepancies. If a discrepancy could not be resolved between the two reviewers, KG reviewed the discrepancy and made a decision. Subsequently, the remainder of the articles underwent data extraction, and AC and GD discussed the results, resolved discrepancies through discussion, and compiled the results. The following data were extracted for each article in the Google Form: study title, author, country of origin, year of publication, study type, population studied, sample size, objective, methods, intervention, comparator, duration of intervention, and outcome(s). The form also included key findings for THN program characteristics including naloxone availability and dispensing patterns, barriers, facilitators, and knowledge gaps about community pharmacy-based THN programs.

As this is a scoping review, we did not conduct a quality appraisal, which is consistent with the framework proposed by Arksey and O’Malley’s methodological guidance for scoping reviews [16]. Results from the data extraction pertaining to study objectives and key findings were revealed through a narrative summary and thematic charts that distribute the objectives across the identified studies. The narrative summary also identified the types of program interventions, naloxone dispensing patterns, and when evaluation of pharmacy-based THN programs took place.

## 3. Results

**Figure 1:**
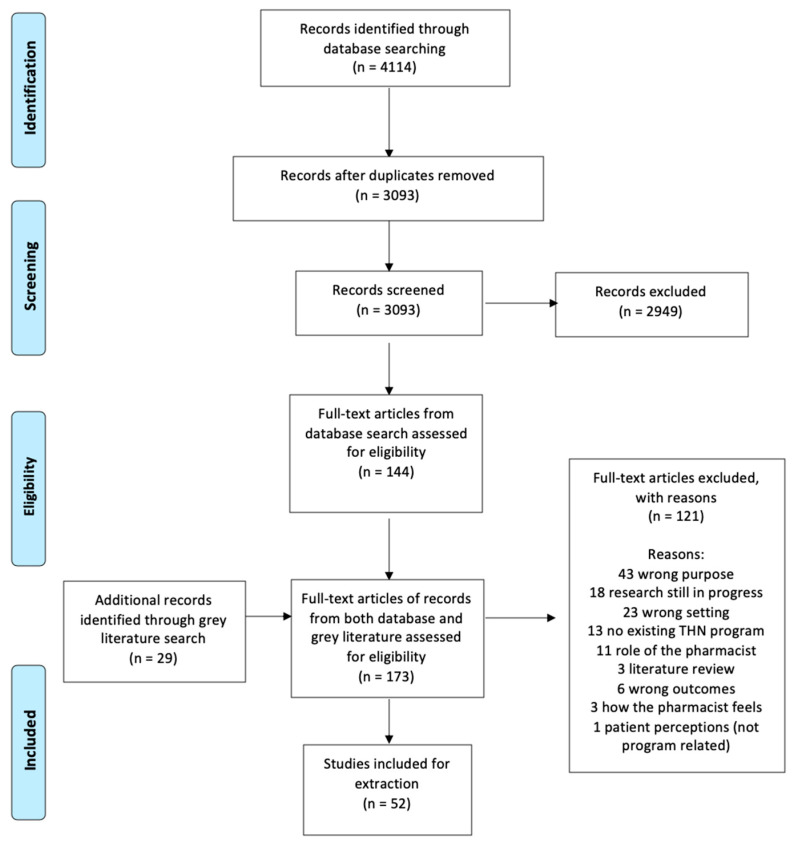
Flow diagram of included studies (THN: Take-Home Naloxone) [17].

A total of 4114 articles were obtained from searching the databases, PubMed, EMBASE, Scopus, and IPA. After deduplication, 3093 articles were screened, and 173 full-text articles, inclusive of 29 grey literature full-text articles, were reviewed. A total of 52 articles were included (Figure 1), including 18 intervention/implementation-type studies (Appendix C) and 34 non-intervention studies (Appendix D). The majority of the included sources were either intervention (12/52, 35%) or cross-sectional survey (19/52, 37%) studies. No randomized controlled trials were identified. See Table 1 for the distribution of sources by scoping review objective.

### 3.1. Take-Home Naloxone Programs and Program Interventions

Eighteen out of 52 studies (35%) described the implementation of a community pharmacy-based THN program (Appendix C). Almost all of the intervention studies (16/18, 89%) were conducted in the United States, though the earliest reported THN program was from 2014 in Scotland [18]. No differences between countries and program types were discovered as there was not a wide variety of countries identified in the review. Most studies were published between 2019 and 2020. Ten out of 18 studies (55%) measured the impact of the THN program on naloxone dispensing; all noted that naloxone dispensing increased after THN program implementation [19,20,21,22,23,24,25,26,27,28]. Patient outcomes were not commonly studied; however, some studies (4/18, 22%) discussed the efficacy of the THN program through opioid overdose reversal rates or the number of naloxone kit refills [18,29,30,31]. There was a wide variety of programs which can be grouped under the following themes: laws, opt-out dispensing, education of pharmacy teams, and interventions such as screening tools, checklists, and pocket-guides (Table 2). The following paragraphs describe key examples of some THN programs.

### 3.2. THN Programs: Laws

Some programs used laws to enhance naloxone access, such as expanding the pharmacist’s naloxone prescriptive authority [19,21,24], making naloxone an over-the-counter medication [30], or mandating that naloxone be co-prescribed with an opioid prescription [27]. For the latter study, the program was implemented across several US states and mandated naloxone as a co-prescription to patients with specific criteria [27]. In some states, a naloxone kit was co-prescribed with any opioid prescription, whereas in other states, it was co-prescribed with patients taking more than 90 Morphine Milligram Equivalents (MME) per day [27].

### 3.3. THN Programs: Opt-Out Dispensing

One study described an opt-out naloxone dispensing program, where each patient presenting to the pharmacy with an opioid prescription written for greater than or equal to a daily dose of 50 MMEs was prescribed and dispensed naloxone by the pharmacist [25]. If the patient declined naloxone during counselling, the pharmacist provided a patient handout and documented the interaction in the patient profile [25]. A noted benefit for this program style was that naloxone was pre-dispensed and did not prolong wait times for patients [25]. The study noted that patients appreciated the naloxone recommendations even if they decided to not take the naloxone kit [25].

### 3.4. THN Programs: Education of Pharmacy Teams and Screening Tools

Several studies incorporated pharmacy team training ahead of implementing various interventions such as patient screening tools [32,33]. Two studies described the Opioid and Naloxone Education (ONERx) program, which involved trained pharmacists screening patients who present with an opioid prescription, for the risk of opioid misuse and accidental overdose [32,33]. Prior to implementing ONERx, pharmacy staff completed a training program to familiarize themselves with the screening process [32,33]. The screening is completed by each patient before receiving an opioid medication, and upon reviewing the results, pharmacists provide patient-specific education and interventions using a clinical decision-making triage tool [32,33]. Some of the pharmacist interventions implemented under this program were: partially filling the opioid prescription, discussing community support services, opioid use disorder and accidental overdose, as well as explaining the benefits of naloxone, and dispensing a naloxone kit [32,33].

### 3.5. THN Programs: Checklists and Pocket-Guides

The first THN program in Washington coupled naloxone dispensing with a standardized patient naloxone counselling checklist [29]. Programs implementing pocket-guides for pharmacists or patients have also been described [20,23]. One THN program used pharmacy claims data to identify patients at high risk of opioid overdose and provided those patients with an Opioid Safety Guide (OSG) during the dispensing of their next opioid prescription [20]. The odds of a patient with an OSG receiving naloxone were four times higher compared to the control [20]. Another program used a pocket card reference for pharmacist communication techniques, which emphasized how to start conversations about naloxone with patients, and provided the counseling points and potential patients who would benefit from a naloxone kit [23]. The implementation of this communication pocket card reference resulted in a four-fold increase in pharmacist naloxone dispensing, when compared with the prior 3 months before pocket card implementation [23]. There have been a number of different interventions applied at various points in the pharmacy workflow as described above, and it is evident that all positively impact THN dispensing numbers.

### 3.6. Naloxone Availability and Dispensing

Multiple sources studied the availability of naloxone and dispensing patterns (22/52, 42%) (Appendix C and Appendix D). Despite the success for the previously mentioned THN programs, several sources found that few pharmacists ever offer or dispense naloxone, despite stocking it in their pharmacies or existing standing orders [11,12,36,37,38,39,40]. For example, a survey of 284 community pharmacists in Indiana found that over half (58.1%) of pharmacies stocked naloxone, but only 23.6% of pharmacists ever dispensed it at the pharmacy [39]. Naloxone availability in community pharmacies also varies significantly despite existing standing orders for pharmacist prescriptive authority or the removal of naloxone from the prescription drug list [11,40,41,42,43]. This makes accessing a life-saving medication difficult, especially since sources found that naloxone is less likely to be available in districts with high rates of opioid overdose deaths, minority neighborhoods, and low household income [38,41,44,45].

The disparity in accessing naloxone was worse in rural pharmacies and independent pharmacies, which the studies found to be less likely to stock and dispense naloxone when compared to urban and chain community pharmacies [12,38,39,40,42,46,47,48,49,50]. In an Ontario population-based study, only 13.1% of naloxone dispensing pharmacies were in rural areas [12]. Meyerson et al. found that chain pharmacies were 3.2 times more likely to stock naloxone, when compared to independent pharmacies [39]. Therefore, these sources show that access to naloxone varies considerably by pharmacy type, region, and within districts, where the populations that need naloxone the most do not have access to it. When a naloxone kit is dispensed, a study examining pharmacy dispensing data found that the most common reason for dispensing naloxone was the patient’s request, and 89.5% of patients indicated that it was their first naloxone kit [47]. This demonstrates that many patients could benefit from a naloxone kit if pharmacists proactively offered them, instead of waiting for patients to request one. Other sources identified that the specific factors associated with increased naloxone stocking or dispensing include having more than one full-time pharmacist on staff [39], a higher opioid prescription volume [51], higher syringe sales [51], and a younger patient population [51].

### 3.7. Facilitators and Barriers

Thirty out of 52 studies (58%) reported facilitators or barriers (Appendix C and Appendix D). The top three barriers reported were (1) concerns over naloxone cost or insurance coverage (12/30, 40%), (2) stigma associated with naloxone from both the patient and pharmacist perspective (12/30, 40%), and (3) the need for further education or training for the pharmacist (9/30, 30%). For a summary of all facilitators and barriers distributed by sources, see Table 3.

### 3.8. Barriers: Cost

For patients without drug insurance, limited drug coverage, or in areas where naloxone is not publicly funded, the median price of USD 145 for intranasal naloxone can be a burden for access [41]. Taylor et al. demonstrated this when they screened patients for naloxone eligibility and provided free naloxone kits [26]. Dispensing rates improved by 163% after the first 3 months of using the screening tool and providing free naloxone kits compared to the prior 3 months [26]. This barrier for patient access is lifted once naloxone kits are offered for free to patients [26].

### 3.9. Barriers: Stigma

Stigma from both the patient and pharmacist perspectives was mentioned. From the patient perspective, patients reported not feeling comfortable asking for naloxone because they were afraid of future consequences [52]. This includes fear of potential change in the pharmacist’s attitude towards the patient if they were to ask for a naloxone kit, or fear of any consequences of being labelled as an “addict” for having a history of naloxone dispensing in their patient profile [52]. From the pharmacist’s perspective, pharmacists reported being afraid of offending a patient when offering them a naloxone kit [52,53]. For example, in Olsen et al., some pharmacists mentioned that they were uncomfortable introducing the subject of naloxone with patients whom they felt were at risk of opioid overdose [53]. The pharmacists stated that they were not sure how to introduce the topic of opioid overdose and naloxone to patients taking prescription opioids as they felt that it would make the patient feel judged [53]. As a means of reducing stigma, one patient suggested having store policies for pharmacists universally offering naloxone, rather than always waiting for the patient to initiate the conversation [52]. Another solution to reduce stigma and increase privacy associated with naloxone was to have naloxone pamphlets available for patients to present to a pharmacist to reduce the need of verbally asking for a naloxone kit [52]. From the pharmacists’ perspective, another solution to reduce stigma would be to increase advertising and marketing of naloxone inside pharmacies, to raise public awareness of the benefits and to increase patient comfort in asking for a naloxone kit [54]. While some pharmacists produced solutions for overcoming stigma, others still endorsed negative beliefs about naloxone, such as that it allows for riskier opioid use and that it allows people who use opioids illicitly to avoid seeking substance use treatment [46]. Therefore, stigma remains a major barrier on both the patient and pharmacist parts for obtaining naloxone.

### 3.10. Barriers: Education and Training for Pharmacists

Education was another of the top three barriers identified. Some studies made recommendations for future educational topics and others mentioned pharmacist-perceived knowledge gaps [36,40,55]. These are further discussed in the “Knowledge Gaps” section, where potential areas of training interest were noted by pharmacists [55]. In addition to the top three mentioned barriers, other common barriers from the pharmacist perspective include a lack of patients asking for naloxone, concerns in pharmacy workflow, lack of time, lack of support from management, and patient refusal (Table 3) [11,24,25,54,55,56,57].

### 3.11. Facilitators

Although less frequently mentioned, there were a variety of facilitators identified in the studies (Table 3). These included education and training for both pharmacists and patients, standardization of training, comfort in discussing naloxone, and interprofessional collaboration [25,36,37,48,54,56,57,58,59]. Pharmacists noted how practice and experience in dispensing naloxone with the opt-out model made it easier to perform [25]. Another study found that a facilitator was additional training for pharmacists on how to initiate conversations with patients [57]. Resources to increase awareness of naloxone access without a prescription were another identified facilitator [37]. One intervention study had an opt-out model for naloxone prescribing, where one pharmacist at each intervention pharmacy prescribed naloxone [25]. At the end of the study, participants remarked that, within a pharmacy, having all of its pharmacists able to prescribe naloxone (as opposed to just one) would facilitate provision [25]. Two studies spoke to the notion of standardized training as a facilitator to naloxone provision [56,59]. The first explained the importance of having standardized state-mandated training requirements for naloxone-dispensing pharmacists to increase naloxone provision [59]. The other study interviewed pharmacy leaders [56]. Chain pharmacy leaders mentioned the importance of standardized training to ensure that pharmacists know that naloxone can be dispensed to more than just illicit drug users [56].

### 3.12. Knowledge Gaps

Knowledge gaps were found for both patients and pharmacy professionals (17/52, 33%) (Appendix D, Table 4). The following themes were identified: misinformation, awareness, administrative, clinical, and counselling (Table 4). For patient misinformation gaps, the most common was the belief that since the patient had been taking a chronic opioid prescription for a long time, then neither they nor their family members were at risk of an opioid overdose [25,28,61]. Another study found that patients held the belief that only people who use substances illicitly needed naloxone [52]. The same study identified that patients were unaware that pharmacists could provide naloxone, demonstrating an awareness gap [52].

For pharmacist knowledge gaps, five studies highlighted administrative knowledge gaps in dispensing requirements [30,45,52,53,61]. One of these studies was an interview of patients, who noted that it was apparent that the pharmacy staff lacked training around naloxone dispensing and billing procedures [61]. Three studies showcased pharmacists not being knowledgeable of the standing order under which they could provide naloxone [36,40,41]. In other words, pharmacists were not aware that they were able to prescribe and dispense naloxone independent of a physician [36,40,41]. For example, in a study where survey interviewers posed as customers, some pharmacy staff incorrectly stated that a prescription from a physician was required for access to naloxone [40]. Other studies identified clinical knowledge gaps such as the need for improvement in methods for initiating discussions with patients about naloxone [36,53,55,57]. Four studies found that pharmacists hold the belief that naloxone increases riskier opioid use practices and overdoses [46,53,57,66]. In addition to this belief about naloxone, a telephone survey found additional negative beliefs held by pharmacists, including the notion that it allows opioid users to avoid seeking substance use treatment and avoid emergency treatment after an overdose [46]. Another two studies demonstrated a lack of understanding of cases in which naloxone is clinically appropriate [45,63]. Both showcased a lack of understanding with respect to the existence of a minimum age requirement [35,45], while one of them further demonstrated confusion regarding naloxone’s appropriateness in pregnancy or in patients who are undergoing an alcohol or barbiturate overdose [63]. Counselling knowledge gaps existed for topics such as when to call 9-1-1 when witnessing an overdose and placing the patient in the recovery position [63].

Several studies had pharmacists make recommendations for future educational topics to address knowledge gaps. Examples of mentioned topics included methods for naloxone counselling, standing orders, choosing between formulations, and identifying patients who would benefit from naloxone [36]. Another survey of pharmacists noted that areas of training interest were strategies in initiating patient discussion, identifying eligible naloxone patients, implementing a naloxone program into the pharmacy workflow, and triaging patients to other resources when appropriate [55]. Therefore, multiple knowledge gaps and previous barriers mentioned could be resolved through targeted training that addresses these and provides pharmacists with the confidence and comfort to proactively offer and dispense naloxone.

## 4. Discussion

This review scoped the available literature on what is known about community pharmacy-based THN programs. This review identified that a lot of research on implementing and evaluating THN programs including program interventions has been published recently, demonstrating that this is a new and rapidly progressing research area. While a wide variety of THN programs were described, none measured patient outcomes and few measured program efficacy [18,29,30,31]. Of the programs that were evaluated, they all showed a positive impact on naloxone dispensing [19,20,21,22,23,24,25,26,27,28]. This review involved research in Canada, the US, Australia, and Scotland. Other countries who function similarly to these could implement similar pharmacy THN programs. Such countries would be able to use the findings in this review to improve or build new THN programs. As no randomized controlled trials were identified, this remains a gap where the THN programs described in this review could be studied in larger populations in a systematic way to see which of the programs identified are more effective and provide positive patient outcomes.

This review also identified that naloxone availability varies significantly by pharmacy type, region, and within districts [12,38,39,41,44,45]. Rural, independent pharmacies and districts with lower household income, increased rates of opioid overdose deaths, and minority neighborhoods are less likely to have naloxone available [12,38,39,41,44,45] This may be because some patients might not have health insurance to cover the naloxone, and poorer cities might not create the demand for pharmacies to stock naloxone [44]. This strengthens the argument for ensuring naloxone is publicly funded everywhere so that such disparities do not happen. Compared to independent pharmacies, chain pharmacies had higher numbers for the dispensing or stocking of naloxone [21,38,39,49,50]. Given the ready access of resources for chain pharmacies, this is a reason for the high likelihood for naloxone availability and high response to the implementation of standing orders [21]. In addition, some chain pharmacies acknowledge having policies and procedures in place regarding naloxone [21]. Therefore, ways to increase naloxone availability to the above mentioned populations need to be investigated, so that naloxone is getting into all of the hands that need it the most.

Multiple barriers and facilitators were identified. Cost as a barrier for naloxone dispensing could be lifted through public funding of naloxone, especially in the cases of low household income [26]. Stigma associated with naloxone and people who use opioids illicitly was identified multiple times but was not extensively discussed in other literature reviews in this research area [13,15]. Education for both pharmacists and the public is needed to close knowledge gaps around myths and misinformation about naloxone—for example, whether or not naloxone increases riskier opioid use [46]. Stigma could be potentially reduced through pharmacist education that incorporates the normalization of naloxone in THN programs [52]. Further training should also cover knowledge gaps found for administrative and clinical issues found in this review, where pharmacists are unfamiliar with standing orders, billing procedures, initiating conversations, and identifying eligible patients [36,53,55,61]. The communication technique incorporated with a THN program that was identified in this review provides a great example of how education and training can help to fill knowledge gaps and lift barriers such as stigma, to increase naloxone availability [23].

This review adds to the literature because it showcases all of the most recent studies on THN programs and program interventions since Clark et al.’s systematic review from 2014 or Nielson’s review from 2016 [10,13]. This is significant as the majority of the studies identified in this review were from 2019 and 2020. This review also provides a different perspective where the focus was not to describe program efficacy, as in Clark et al.’s systematic review [10], but rather to showcase the types of programs being implemented currently so that they can be carried out in randomized controlled trials or large-scale studies that measure efficacy and patient outcomes. While Nielson and Thakur et al.’s literature reviews discussed facilitators and barriers for naloxone dispensing [13,15], this review adds to the literature because it highlights and discusses multiple barriers but, more importantly, expands on stigma as a barrier and discusses solutions for how to overcome stigma from both the pharmacist and patient perspectives. In fact, other reviews, such as Thakur et al., discussed how more research was needed to highlight how de-stigmatization could be implemented in community pharmacies, which has been discussed in this review in the form of program interventions [15]. This review adds to the literature because it highlights examples for how to overcome barriers such as stigma through the discussion of program interventions including communication techniques and brochures and the key topics with solutions that should be addressed in future pharmacist training modules to overcome barriers such as stigma and knowledge gaps.

This study does not come without its limitations. First, sources were only included if they were in English or French. Therefore, this review may have missed important international differences if sources were published in other languages. Second, multiple sources were not included as the research was still in progress and results could not be identified. This could contribute to the review missing potentially important findings. Third, while this review was aimed to be extensive, there is a possibility that not all work was identified by the search or databases used. Lastly, no quality assessment was conducted as the focus was to cover the range of work that informs about THN programs, rather than limit to a select few that meet quality assessment standards. There were no deviations in the review protocol from the Arksey and O’Malley framework or the PRISMA guidelines [16,17].

## 5. Conclusions

Implementing and evaluating community pharmacy-based THN programs is a rapidly progressing research area. Multiple small-scale studies have described a variety of THN program interventions that could be evaluated through a randomized controlled trial for efficacy and patient outcomes. Due to the fact that naloxone availability varies, a targeted standardized education program should be developed for pharmacists that implements strategies to overcome barriers, particularly stigma, and knowledge gaps that were identified in this review. Future research in this area should (1) be intentional in conducting interventions in line with THN programs, (2) be rigorous in study design, including randomized controlled trials, (3) measure program efficacy and patient outcomes, and (4) incorporate pharmacist training into programs that target the barriers and knowledge gaps mentioned in this review.

## Figures and Tables

**Table 1:** Distribution of sources by study objective.

Objective	Number of Studies (%)
Take-Home Naloxone Programs	18 (35%)
Naloxone Availability and Dispensing	22 (42%)
Facilitators and Barriers	30 (58%)
Knowledge Gaps	17 (33%)

**Table 2:** Distribution of Studies for each type of THN program identified (*n* = 18).

Theme	Number of Intervention Studies (%)
**Laws**	
Mandatory naloxone co-prescription with opioids	1 (5.6%) [27]
Standing order	2 (11.1%) [21,24]
Prescriptive authority	4 (22.2%) [19,21,24,31]
OTC status of naloxone	1 (5.6%) [30]
Education of pharmacy team prior to implementation	10 (55.5%) [18,22,23,24,29,31,32,33,34,35]
**Interventions**	
PMS used to help flag patients eligible for naloxone	3 (16.6%) [28,34,35]
Checklists (patient eligibility, patient counselling)	4 (22.2%) [22,25,29,34]
Pocketcard	1 (5.6%) [23]
Patient brochure	4 (22.2%) [20,22,34,35]
Screening	4 (22.2%) [26,32,33,34]
**Opt-out**	1 (5.6%) [25]

**Table 3:** Distribution of studies for barriers and facilitators (*n* = 30).

Barriers or Facilitators	Number of Studies (%)
**Top Barriers**	
Cost/coverage	12 (40%) [11,24,26,35,41,44,45,46,47,55,56,57]
Stigma	12 (40%) [11,28,45,46,47,52,53,56,58,60,61,62]
Education/training for pharmacist	9 (30%) [11,35,36,40,47,55,57,61,63]
**Other Barriers**	
Lack of patients asking for naloxone	4 (13.3%) [11,24,37,46]
Patient refusal	2 (6.6%) [25,45]
Workflow concerns	3 (10%) [33,55,56]
Lack of time/space	5 (25%) [33,35,36,52,56,57]
Lack of support from management	2 (6.6%) [55,57]
Pharmacy reimbursement/remuneration	5 (25%) [24,46,48,55,57]
**Facilitators**	
Pharmacist education/training	5 (25%) [36,45,48,57,59]
Patient education/training	2 (6.6%) [45,57]
Standardization in training	2 (6.6%) [56,59]
Comfort discussing naloxone	3 (10%) [36,37,58]
Interprofessional collaboration	3 (10%) [25,56,58]
Patient private counselling	1 (3.3%) [58]
Not asking for identification	1 (3.3%) [64]
Pharmacy protocols	1 (3.3%) [65]

**Table 4:** Distribution of studies for knowledge gaps (*n* = 17).

Knowledge Gap	Number of Studies (%)
**Patient Knowledge Gaps**	
**Theme: Misinformation**	
Belief that only people who use substances illicitly need naloxone	1 (5.8%) [52]
Belief that since they have been taking chronic opioid prescriptions for awhile either them or their family members are at risk of opioid overdose	3 (17.6%) [25,28,61]
**Theme: Awareness**	
Unaware pharmacies can provide naloxone	1 (5.8%) [52]
**Pharmacist Knowledge Gaps**	
**Theme: Administrative Gaps**	
Standing order	3 (17.6%) [36,40,41]
Triaging patients to other resources when appropriate	1 (5.8%) [55]
Dispensing requirements	5 (29%) [30,40,45,52,53,61]
**Theme: Clinical Gaps**	
Understanding of the purpose of naloxone	1 (5.8%) [53]
Belief that naloxone increases riskier opioid use practices and overdoses	4 (23.5%) [46,53,57,66]
Specific circumstances of when naloxone can be given (ex: alcohol/barbiturate overdoses, in minors, in pregnancy)	2 (1.2%) [45,63]
Ability to identify eligible patients	4 (23.5%) [18,36,55,58]
Knowledge of different formulations	2 (1.2%) [36,63]
**Theme: Counselling Points**	
Course of action after administering naloxone	1 (5.8%) [36]
Calling 9-1-1 when witnessing an overdose	1 (5.8%) [63]
Starting rescue breathing	1 (5.8%) [63]
Placing patient in the recovery position	1 (5.8%) [63]
Methods for teaching/starting discussions with patients about naloxone	4 (23.5%) [36,53,55,57]

## Data Availability

Not Applicable.

## Appendix A

### EMBASE Search Strategy (Performed 1 June 2020)

1. exp “pharmacy (shop)”/
2. exp pharmacist/
3. (Pharmacist* or Pharmacy or Pharmacie* or Pharmaceutical service*).mp. [mp=title, abstract, heading word, drug trade name, original title, device manufacturer, drug manufacturer, device trade name, keyword, floating subheading word, candidate term word]
4. 1 or 2 or 3
5. exp narcotic antagonist/
6. exp opiate antagonist/
7. (Naloxone or Narcan or Naloxon or Nalone or Antidote).mp. [mp=title, abstract, heading word, drug trade name, original title, device manufacturer, drug manufacturer, device trade name, keyword, floating subheading word, candidate term word]
8. 5 or 6 or 7
9. exp drug overdose/
10. exp harm reduction/
11. exp counseling/
12. exp drug dependence/
13. exp opiate addiction/
14. exp drug abuse/
15. exp narcotic dependence/
16. (Addiction or Counsel* or Abus* or Dependenc* or Overdos* or Death* or Harm* or Naloxone distribution or Program* or Safe* or Poisoning or Preventative Health Service* or Substance abuse or Take home or Take-home or THN or Train* or Prevent*).mp. [mp=title, abstract, heading word, drug trade name, original title, device manufacturer, drug manufacturer, device trade name, keyword, floating subheading word, candidate term word]
17. 9 or 10 or 11 or 12 or 13 or 14 or 15 or 16
18. 4 and 8 and 17

## Appendix B

**Table A1:** Data extraction form sample.

Study Name
Author
Year
Study type
Country
Objective(s)
Population studied
Sample size
Methods
Intervention/comparator
Duration of intervention
Outcomes studied
Key Findings: Facilitators/barriers for existing THN programs
Key Findings: THN program details
Key Findings: Knowledge gaps about THN programs

## Appendix C. Intervention Studies

**Table A2:** Intervention studies (*n* = 18).

Author	Year	Location	Population Studied	Methods	Intervention and Comparator	Outcomes Studied	Key Findings
**Cohort studies**							
Gangal [19]	2020	USA	Patients	Cohort study	I: Pharmacist prescriptive authority C: Baseline	Naloxone dispensing rate	- Naloxone dispensing rate/month/county increased significantly. - Patients in low income or high poverty areas were significantly more likely to obtain naloxone post-policy.
Champaloux [20]	2018	USA	Patients at high risk of overdose	Prospective cohort study	I: Opioid safety guide (OSG) opioid dispensing C: Non-OSG opioid dispensing	- Naloxone dispensing rate - Opioid discontinuation	- The OSG included education about safe opioid use, disposal, storage, and naloxone. - The odds of a patient receiving naloxone were 4 times higher compared to control.
**Mixed Methods**							
Eldridge [21]	2020	USA	Pharmacies	Looking at pharmacy censuses, naloxone stocking/dispensing rates, naloxone policies, and educational programs	I: Statewide standing order C: Baseline	Naloxone stocking and dispensing	- Naloxone dispensing rates tripled between 2016 and 2018. - Corporate education efforts have the potential to have an impact on stocking and dispensing; chain drug stores were more likely to dispense and stock naloxone.
**Intervention and Implementation Study**							
Akers1 [29]	2017	USA	N/A	Implementation study	I: Pharmacist training and dispensing checklist	Implementation and early results of the first THN program in a community pharmacy in Washington	- Education program coupled with dispensing checklist to standardize patient counselling including risk factors for and recognizing opioid overdose, providing basic life support, naloxone administration, recovery position, post-overdose monitoring, and Good Samaritan law review. - The main population accessing the program are family/friends and are generally older population.
Wilkerson [34]	2020	USA	N/A	Implementation study	I: Pharmacist training and patient counselling checklist	Implementation of a naloxone dispensing program in which pharmacists and pharmacy interns (under the direct supervision of a pharmacist) can dispense naloxone without a prescription.	- All pharmacists and interns completed a training program about overdose prevention and naloxone rescue kits, naloxone resources, patient education checklist form, and a training video. - To dispense, eligible patients are identified or can ask for naloxone. - If identified, the patient fills a consent form with qualifying information, which acts as a prescription. - A note is added to the pharmacy software serving as a hard-stop. - At pickup, pharmacy staff reviews the checklist form, counsels, provides naloxone and a brochure. - The pharmacist must sign off that counselling has been completed, and documentation is scanned into pharmacy software.
Freeman [30]	2017	CND	N/A	Implementation study in which a number of groups worked together to coordinate policy changes	N/A	- Implementation of Alberta’s THN program - Number of sites distributing naloxone that were pharmacies - Ease of naloxone access at pharmacies	- From 2015 to 2016, THN kits were dispensed through 953 registered sites, 759 of them community pharmacies. - Initial feedback showed there was variation in accessing THN kits. - To support assessment of the program, people who use a THN kit are asked to call a toll-free nursing line to report use of kits and outcome. - Some pharmacies unaware that people could access kits without providing a provincial healthcare number.
Shafer [22]	2017	USA	Walgreen’s pharmacies	Intervention study employing 3 programs at Walgreens evaluated using the RE-AIM framework	I: 3-pronged approach taken by Walgreens: (1) provide safe medication disposal kiosks (2) expand national access to naloxone (3) provide education on the risk and avoidance of opioid overdose.	Naloxone prescriptions dispensed How naloxone-prescribing policies in select states have positively affected naloxone dispensing through Walgreens	- Early results are safe medication disposal kiosks in more than 43 states, naloxone-dispensing program in 33 states. - The naloxone training program is required for all pharmacists. - It includes policies, risk factors for, and recognition of overdose; how to use naloxone products; procedures for handling an overdose; and the impact of overdose in the USA. - The pharmacist counsels every patient who is receiving naloxone and provides them with written educational materials.
Gandhi [23]	2020	USA	Pharmacists	Intervention study	I: Communication technique training (CTT) C: Baseline before training	Dispensing rates of naloxone and pharmacist and pharmacy-related factors that may affect the dispensing rates of naloxone	- The CTT consisted of a pocket card reference and patient-case scenario activity. - The cases emphasized the points found on the card and were taught as a teach-back method. - The CTT caused 4-fold increase in naloxone dispensing.
Morton [24]	2017	USA	Pharmacies	Intervention study with a top-down approach of expanding legislation to include a standing order for naloxone and a bottom-up approach of pharmacy staff training	I: Expanding legislation by allowing standing order for naloxone and with pharmacy staff naloxone training C: Dispensing patterns of same pharmacies in previous years	Number of: - pharmacies dispensing naloxone - naloxone doses dispensed - counties in which naloxone was available through pharmacies	**Barriers**: pharmacy reimbursement, affordability for patients, and lack of patient interest. - 9-fold increase in the first half of 2016 of naloxone Medicaid claims over 2014. - The bottom-up approach included pharmacy staff training, free resources for pharmacies, and peer-to-peer coaching/support. - The Human Services Department initiated a public awareness campaign to promote opioid overdose death and naloxone awareness.
Skoy [25]	2019	USA	Patients	Intervention study at three pharmacies	I: Opt-out naloxone dispensing C: None	-Implementation of an opt-out naloxone dispensing program - Patients prescribed and dispensed naloxone	**Barriers**: lack of patient perceived need due to chronic opioid use without problems **Facilitators**: having all pharmacists working authorized to prescribe naloxone, not labeling the naloxone kit until the patient accepted the service, involving technicians in the training process, automatic calculation of Morphine Milliequivalents (MME)/day within the dispensing software - Naloxone nasal spray was most commonly prescribed. - Each patient presenting to the pharmacy with an opioid prescription written for ≥ 50 MME/day was prescribed and dispensed naloxone by the pharmacist if not already on their profile. - If prescribed and dispensed, a form called “Naloxone Worksheet” was attached to the final prescription bag to serve as a hard-stop. - Even if a patient denies naloxone, a handout on naloxone was provided. Moreover, if refused, the naloxone stays on file for future review. - Pharmacists reported increased comfort in recommending and dispensing naloxone. -RPhs mentioned that technicians should be involved in the training process since they are typically involved at intake and point of sale.
Strand [32]	2019	USA	Pharmacies	Intervention study employing a statewide risk assessment program called ONE Rx	N/A	-Implementation of the ONE Rx program - Populations impacted by the program - Future implications of the program	- The program involves screening patients who receive an opioid prescription for the risk of opioid misuse and accidental overdose and offering interventions which can include discussing benefits of naloxone and offering a naloxone kit. - The screening was completed by each patient before receiving an opioid medication. - Based upon screening results, pharmacists provided education and interventions using a clinical decision-making triage tool. - Pharmacist interventions with included: opioid prescription partially filled, discuss community support services, explain benefits of naloxone, dispense naloxone, discuss opioid use disorder, discuss accidental overdose.
Taylor [26]	2020	USA	Patients	Intervention study at a community pharmacy within a healthcare clinic serving low socioeconomic status patients	I: Naloxone screening tool (3 months) C: Baseline (previous 3 and 12 months)	Naloxone dispensing	**Barriers**: availability of naloxone, cost, pharmacist lack of comfort with naloxone - Survey consisted of 3 questions that were read to patients upon each visit. - Those deemed eligible for naloxone received a naloxone training session along with a free kit. - Dispensing rates of naloxone improved with active identification of patients who qualify or need access to naloxone, despite active naloxone advertisement in the pharmacy. - Providing naloxone free of cost lifted barriers for patients.
Bachyrycz [31]	2015	USA	Pharmacists	Intervention study evaluated using a brief reporting form	I: Pharmacist training and naloxone dispensing	Trends in opioid overdose prevention	- Majority of the patients accessing naloxone noted it was their first time. - Most common reason for naloxone prescription was due to high dose prescription opioids, followed by chronic opioid use. - Request for naloxone by a patient was primarily from patients on opioid maintenance therapy.
Green [27]	2020	USA	CVS Pharmacies	Intervention study	I: Naloxone mandated co-prescription C: Standard of practice	Pharmacy naloxone provision	- Naloxone-prescribing mandates tripled pharmacy naloxone provision - This approach engaged more prescribers, complemented ongoing naloxone provision under pharmacy standing orders, expanded geographic reach, and broadened the naloxone payor mix.
Griffin [28]	2019	USA	Patients on Opioid Agonist Therapy, chronic opioid therapy, high-risk, or >= 50 MME/day	Intervention study	3-month pre-intervention period 1-month staff training intervention 3-month post-intervention period.	Naloxone prescriptions dispensed	**Barrier**: Stigma (not wanting to be labeled an addict) - Patients were tagged in the pharmacy software if they met inclusion criteria and then guided toward the pharmacy consultation room when they presented at the pharmacy. - Patients did not think they needed naloxone because they had been taking opioid medications for years or they had stopped their opioid prescription.
Skoy [33]	2019	USA	Pharmacies	Intervention study employing a statewide risk assessment program called ONE Rx	I: The ONE Rx program C: None	Number of: - Patients screened for opioid misuse and accidental overdose with the ONE Rx program -Pharmacists/technicians who participated in the training - Pharmacies who chose to implement ONE Rx	**Barriers**: Time, workflow - Pharmacy staff completed a training, and then implemented the program through screening patients at risk of opioid misuse and accidental overdose. - Every patient receiving an opioid prescription is screened for the following: risk of opioid use disorder using Opioid Risk Tool and risk of accidental overdose based on age, concurrent medications, and disease states. - The patient completes a screening tool before receiving an opioid medication. - Pharmacist reviews the screening results and provides patient-specific education and interventions using a clinical decision-making triage tool - Potential interventions can include counselling on (1) dispensing of naloxone, (2) substance use disorder, (3) referral to community support services, (4) partial fill of opioid prescription, (5) and providing pharmacy medication disposal.
Sexton [35]	2019	USA	Patients at risk of opioid overdose	Intervention study	I: Standardized team-based approach to identify naloxone-eligible persons. C1: Standard practice at control store. C2: Previous year dispensing numbers at both stores.	- Naloxone kits dispensed - Access to naloxone - Pharmacist–patient relationship	**Barriers**: cost - The program consisted of the pharmacy receiving training, a MME conversion chart, eligibility checklist, a list of relevant opioids. - Patients were screened for naloxone eligibility during prescription intake, if presented an opioid prescription. - Potential candidates were flagged for the pharmacist and put in the pharmacy software, creating a hard-stop. - All eligible patients—including those who refused naloxone—were provided with an educational handout. - Some patients who accepted a kit reported that they needed to use it and were thankful that they had received a kit.
Laird [18]	2014	Scotland	Injection Equipment Provision (IEP) Pharmacies serving Injection Drug Users	Pilot intervention using pre and post questionnaires to evaluate knowledge	I: Extending provision of staff training and supplies to IEP pharmacies	Feasibility of providing naloxone in community IEP pharmacies	- Injection Equipment Provision (IEP) pharmacies are ideal for offering naloxone training and supply. - None of the pharmacy staff answered the prequestionnaire correctly before training.

## Appendix D. Non-Intervention Studies

**Table A3:** Non-intervention studies (*n* = 34).

Author	Year	Location	Population Studied	Methods	Outcomes Measured	Key Findings
**Cross-sectional survey**						
Shedd [65]	2019	USA	Pharmacists	Emailed survey	Barriers and attitudes	**Facilitator**: pharmacy protocols
Carpenter [26]	2019	USA	Pharmacists	Online survey	Factors associated with how often pharmacists offer and dispense naloxone	**Barriers**: time, inadequate training, perceived lack of patient comprehension **Facilitators**: comfort discussing naloxone, naloxone training for pharmacists - Most pharmacists stocked naloxone, but few offer and even fewer dispense it - Pharmacists working in smaller chain pharmacies dispense/offer naloxone less often than other pharmacy types - Pharmacists request training for: methods naloxone counselling, standing orders, choosing between formulations, and identifying patients who would benefit from naloxone
Graves [40]	2019	USA	Pharmacy staff	Survey with interviewers posing as customers	- Naloxone availability/price - Pharmacy staff knowledge of standing order	**Barriers**: education - National pharmacies are more likely to carry and have knowledgeable staff. - Some pharmacy staff incorrectly stated that a prescription was required for naloxone, or it could not be obtained for a third party.
Guadamuz [41]	2019	USA	Pharmacies	Telephone survey	Availability and cost of naloxone nasal spray	**Barriers**: cost - Naloxone was less likely to be available in districts with elevated rates of opioid overdose deaths, minority neighborhoods. - Despite the standing order, many pharmacists required a prescription.
Egan [49]	2020	USA	Pharmacies	Survey using mystery caller protocol	Pharmacy and neighborhood characteristics associated with pharmacy stocking and willingness to sell naloxone	- An estimated 2 out of 3 North Carolina retail pharmacies have naloxone available without a prescription. - The odds of naloxone availability were lower for independent pharmacies than chains. - Most recommended going to a chain pharmacy for naloxone if it was unavailable at their pharmacy.
Lozo [44]	2019	USA	Pharmacies	Survey with scripted questions	Naloxone availability and sociodemographic factors associated with it	**Barriers**: insurance, cost - Higher median household income indicated more naloxone availability - Cities identified with severe opioid-related public health concerns but limited naloxone access
Spivey [54]	2020	USA	Pharmacies	Telephone survey	- Availability and pricing - Barriers to naloxone dispensing - Strategies to improve access	**Barriers**: cost, patient refusal, lack of insurance coverage, stigma, issues with prescribers, and lack of patient education interest or awareness about naloxone. **Facilitators**: improved insurance coverage, improving naloxone-related education for patients, pharmacists, and other providers - Most pharmacies reported recommending naloxone ≥50% of patients taking an opioid with a dose of 50MMEs/day - For patients co-prescribed benzodiazepines or with respiratory conditions, less than the majority of pharmacies recommended naloxone to ≥50% of that patient population
Nichols [37]	2019	USA	Pharmacists	Online survey	Pharmacist utilization, knowledge, attitude, and barriers to the naloxone standing order	**Barriers**: lack of patients asking for naloxone **Facilitators**: resources to increase awareness of naloxone access without a prescription and pharmacist comfort
Darracq [38]	2019	USA	Pharmacies	Survey	- RPh knowledge of naloxone-dispensing law, participation, and future plans for participation - Naloxone availability - Out of pocket charges to consumers	- Despite high rates of knowledge, current and future participation was low, especially in areas with high rates of prescription opioid-related deaths - Most pharmacies reported naloxone in stock and immediately available. - Counties with higher rates of pharmacy participation were associated with participation by a regional or national pharmacy chain.
Melaragni [63]	2019	USA	Pharmacists	Verbal survey	Pharmacist education and training associated with dispensing naloxone	**Barriers**: Education - Pharmacists demonstrated knowledge gaps for: calling 9-1-1 when witnessing an overdose, starting rescue breathing, placing patients in the recovery position, whether naloxone can be administered for minors or in pregnancy or in alcohol or barbiturate overdoses. - Majority of pharmacists said they would benefit from more training.
Sisson [46]	2019	USA	Pharmacists	Telephone survey	- Pharmacist attitudes toward naloxone - Potential barriers to pharmacy naloxone distribution	**Barriers**: cost, negative beliefs about naloxone, low demand by patients. - Rural pharmacies required more time to obtain naloxone and offered less extensive training. - Independent pharmacies were less likely to stock naloxone, especially rural locations. - Pharmacist negative beliefs about naloxone allow opioid user to conduct riskier practices, avoid seeking substance use treatment, and avoid emergency treatment after an overdose.
Jimenez [45]	2019	USA	Pharmacies	Simulated a routine conversation between pharmacy staff and a potential customer about the immediate availability of and requirements to purchase naloxone using a semi structured questionnaire.	- Availability of naloxone in pharmacies - Pharmacy staff knowledge regarding naloxone dispensing protocols	**Barriers**: lack of availability, ambiguity of the standing order. - Most pharmacies only carry naloxone nasal spray. - In states with highest opioid overdose deaths, about a quarter do not stock naloxone. - Only half of pharmacy employees knew there was no minimum age requirement to purchase naloxone
Kuhn [67]	2019	USA	Pharmacies	Phone survey	- Naloxone availability - Pharmacist familiarity with naloxone standing order, knowledge, and educational compliance	- Most pharmacies had naloxone available and were familiar with the standing order. - Most pharmacies did not have previous knowledge of the Opioid Assistance and Referral Line.
Meyerson [39]	2018	USA	Pharmacists	Census	Factors associated with community pharmacy naloxone stocking and dispensing	- More pharmacists stocked naloxone than dispensed it. - Chain pharmacies significantly more likely to stock naloxone. - Naloxone stocking more likely in pharmacies with ≥1 full-time pharmacist. - Pharmacy manager having naloxone education associated with higher likelihood of pharmacy stocking naloxone.
Bachyrycz [47]	2017	USA	Patients	Data from NRK prescriptions	Prescription patterns of Naloxone Rescue Kits (NRKs)	**Barriers**: optional naloxone certification for pharmacists and variable insurance coverage **Facilitator**: pharmacy accessibility - Most patients indicated it was their first NRK - Most common reason for a NRK was the patient’s request - Few NRK prescriptions given to patients with polysubstance use - NRK program is under-utilized in rural areas
Rudolph [55]	2018	USA	Pharmacists	Online survey	- Barriers to dispensing naloxone - Identify areas for additional training	**Barriers**: discomfort dispensing, inadequate training, workflow concerns, lack of management support, cost/reimbursement, and ethical concerns. - Positive correlation found between pharmacists’ knowledge of naloxone and opioid overdose and willingness to dispense naloxone. - There are no correlations between level of comfort for dispensing naloxone with type of pharmacy practice setting or number of opioids dispensed daily. - Additional training needs included: information regarding naloxone/opioid overdose, strategies to initiate patient discussion, identifying eligible patients, and workflow implementation.
Cressman [11]	2017	CND	Pharmacies	Telephone survey	Availability, cost, and need for a prescription for naloxone	**Barriers**: stigma, cost, perceived lack of demand, pharmacist lack of training. - Availability was highest in BC, followed by the Maritimes, ON and Central and Northern Canada. - QC had the lowest availability. - Some pharmacies who did not carry naloxone referenced not having received the education.
Thornton [66]	2017	USA	Pharmacists	Survey administered at Continuing Pharmacy Education events	- Educational needs relating to naloxone provision in community pharmacies	- Most pharmacists agree that letting patients purchase naloxone OTC will increase opioid overdoses. - Majority of pharmacists are not comfortable selling naloxone without a prescription despite knowing it is effective.
Stone [50]	2018	USA	Pharmacies	Telephone survey	- Availability of naloxone in retail pharmacies	- Independent pharmacies less likely to have naloxone compared to chains.
**Grey literature**						
CDC [42]	2019	USA	N/A	N/A	Naloxone dispensing	- Wide variations in pharmacy dispensing, despite consistent state laws. - Rural counties had the lowest dispensing rates. - One naloxone prescription dispensed per 70 high dose opioid prescriptions
CPhA [43]	2017	CND	Canada	Environmental scan	Naloxone accessibility	- At every purchase, provincial regulations require pharmacists to review a patient’s history of opioid use, naloxone use/response, allergies, and offer counselling. - In all provinces, pharmacists are required to train patients. - Only the Northwest Territories and Yukon have THN kits available in every pharmacy. - The Alberta and Quebec programs enable patients to get a naloxone kit without a health card.
Moustaqim-Barrette [60]	2019	CND	N/A	Environmental scan and conversations with key informants	- Uptake of naloxone programs - Knowledge and operational barriers	**Barriers**: Expiry/temperature of naloxone, stigma in rural areas, minimum training standards, withdrawal/adverse effects **Facilitator**: Political will - The injectable formulation is available in all THN programs. - Ontario, Quebec, and NWT provide nasal naloxone.
Alberta College of Pharmacy [64]	2019	CND	N/A	N/A	Naloxone kits distributed	**Facilitator**: not asking for identification - Identification is not needed when requesting a kit, which reduces stigma. - Improvements are contemplated based on feedback from the naloxone kit usage forms; feedback is only used to improve the program.
BC CDC [48]	2019	CND	N/A	Mixed methods with administrative data and discussions with stakeholders	- Strengths and barriers of THN program implementation and management - Stakeholder attitudes, perceptions, and beliefs	**Barriers**: lack of remuneration, communication gaps, and logistical challenges (lag in data entry) **Facilitators**: modifying implementation procedures (introducing electronic records), refresher training for pharmacists, handouts, intranasal kits - In just one year (2018), over 3000 kits were distributed by 562 pharmacies - Most recipients at pharmacy sites were receiving their first kit - Majority of kits are distributed to individuals not at risk of overdose themselves
Roberts [59]	2019	USA	N/A	Structured internet searches	Naloxone training mandates	**Barrier**: lack of standardized naloxone training requirements for pharmacists - Oregon and Vermont require people who purchase naloxone from community pharmacies to verify that they have completed naloxone education.
**Longitudinal study**						
Green [51]	2019	USA	Pharmacies	Longitudinal analysis of pharmacy naloxone dispensing	Characteristics associated with increased likelihood of naloxone dispensing	Factors associated with an increased likelihood of naloxone dispensing: – rurality - high opioid prescription volume and syringe sales - drive-throughs - long weekend hours - younger patient population
**Population-based study**						
Choremis [12]	2019	CND	Ontario residents	Cross-sectional analysis and descriptive analysis	- Uptake of THN program - Characteristics of the patients and pharmacies	- Only half of Ontario’s pharmacies dispense/offer naloxone. - Highest uptake for patients on opioid agonist therapy. - Minimal uptake for patients on prescription opioids, past opioid exposure, or unknown opioid exposure. - The majority of pharmacies offering naloxone are in urban areas.
**Qualitative studies**						
Green [52]	2017	USA	Patients taking opioids for chronic pain, people with opioid use disorders, caregivers, and pharmacists	Focus groups, semi-structured interviews	Perceptions and experiences of pharmacy naloxone from various groups	**Barriers**: Stigma, patients fearing future consequences, pharmacists fearing offending the patient, time, space constraints. - Practice modification suggested to reduce stigma: pharmacists universally ask patients about naloxone (with some criteria), as opposed to always waiting for the patient to initiate the conversation. - Pharmacists expressed a lack of clarity on logistics, insufficient training and demonstration materials. - Some patients reported believing naloxone was only meant for heroin users and others were unaware that pharmacies could provide it.
Olsen [53]	2019	Australia	Pharmacists	Survey and semi-structured interviews	Pharmacists’ attitudes to and experiences of OTC naloxone.	**Barriers**: low confidence, stigma, limited understanding of opioid overdose, business concerns. - Some pharmacists were unsure of how to advise a patient who requested naloxone, educate patients about naloxone availability, and approach the topic of overdose. - Some pharmacists were unable to correctly state the purpose of naloxone, others had negative views such as attracting an undesirable clientele. - Half of pharmacists were aware that naloxone changed to OTC status.
Donovan [56]	2020	USA	Pharmacy leaders	Semi-structured interviews	Pharmacy leader perception of pharmacies and how staff can optimize naloxone dispensing	**Barriers**: stigma (in general), stigma (within the context of a rural community), comfort, workflow, time, lack of compensation for counselling patients. **Facilitators**: standardized trainings, state regulations and federal initiatives, co-prescribing of naloxone by physicians, pharmacists as public health leaders
Donovan [61]	2019	USA	Obtained naloxone from the pharmacy or high-risk patient as per study criteria	Semi-structured interviews	Factors impacting the likelihood of obtaining pharmacy-based naloxone (PBN)	**Barriers**: Pharmacist education, stigma, disempowerment for patients prescribed opioids. **Facilitators**: Empowerment for people who use drugs. - People prescribed opioids do not feel they are at risk of overdose or need naloxone and feel that their family members are not at risk of an overdose. - Participants also noted a lack of staff training, including confusion about dispensing and billing procedures.
Green [58]	2020	USA	Patients taking opioids for chronic pain, people with opioid use disorders, caregivers, and pharmacists	Focus groups, semi-structured interviews	- Experiences obtaining naloxone at the pharmacy - Reactions to pharmacy tools and patient outreach materials	**Barriers**: Fear of future consequences when requesting naloxone, stigma, discomfort in asking pharmacy staff for naloxone **Facilitators**: practice made pharmacists more comfortable in dispensing, creating privacy for patients, interprofessional collaboration - Participants were accepting of a program that included display pads which patients could present to the pharmacist to reduce the stigma of verbally asking for a naloxone kit. - Pharmacists expressed a desire for further education because they still felt uncomfortable engaging naloxone discussions even with previous experience/training. - Pharmacy technicians lack sufficient program training.
**Mixed Methods**						
Hammett [62]	2014	USA	N/A	Interviews, review of legal and policy documents, and information on stakeholders.	- Feasibility of expanded pharmacy services for people who inject drugs - Legal and policy barriers	**Barriers**: stigma, prescription status - At the time of this study, naloxone was a prescription in all study locations and seldom stocked in pharmacies.
Bakhireva [57]	2018	USA	Pharmacists	Structured, quantitative survey created after focus group	Barriers and facilitators	**Barriers**: cost, time, inadequate pharmacy reimbursement, inadequate privacy for patient counselling, lack of access to full medical records of patients, pharmacy manager does not allow naloxone dispensing, attracting undesirable clientele **Facilitators**: increased awareness among patients about the need for naloxone, education for the general public, and additional training for pharmacists on how to initiate discussions with high-risk patients - Majority of community pharmacists never dispensed naloxone before despite prescriptive authority and standing orders. - Some pharmacists reported fear that dispensing Intranasal Naloxone would promote opioid abuse and attract undesirable clientele to the pharmacy.
